# Acceptability and tolerability of alcohol-based hand hygiene products for elderly residents in long-term care: a crossover study

**DOI:** 10.1186/s13756-019-0610-7

**Published:** 2019-10-29

**Authors:** Margaret O’Donoghue, Jacqueline M. C. Ho, Didier Pittet, Lorna K. P. Suen

**Affiliations:** 1 Squina International Centre for Infection Control, School of Nursing, The Hong Kong Polytechnic University. Hung Hom, Kowloon, Hong Kong; 2 Infection Control Program and World Health Organization Collaborating Centre on Patient Safety, University of Geneva Hospitals and Faculty of Medicine, Geneva, Switzerland

**Keywords:** Hand hygiene, Alcohol-based hand rub, Long-term care facility; Elderly hand hygiene; Tolerability, Acceptability, World Health Organization

## Abstract

**Background:**

Hand hygiene is a critical component of infection control. Much of the focus on improving hand hygiene in healthcare settings has been directed towards healthcare worker compliance but its importance for patients, including those in long-term care facilities (LTCFs), is increasingly being recognised. Alcohol-based hand rub (ABHR) can lead to improved compliance. We aimed to determine acceptability and tolerability of two ABHRs for hand hygiene of elderly LTCF residents using a modified version of the WHO protocol.

**Methods:**

Thirty six elderly LTCF residents participated in this crossover study. A modified and translated (Chinese) version of the WHO protocol for evaluation of two or more ABHRs was used to determine product acceptability and tolerability for one gel (bottle with reclosable cap) and one foam (pump). During the 3-day testing period, participants were provided with their own portable bottle of ABHR. A research nurse objectively assessed the skin integrity of the hands at baseline and throughout the study. Skin moisture content was determined using a Scalar Moisture Checker Probe (Science Technology Resources, Ca, USA). Participants rated ABHR tolerability and acceptability using the WHO checklist at the end of each test period.

**Results:**

Both products passed the WHO criteria for acceptability and tolerability. The foam (86%) scored higher than the gel (51%) for ease of use possibly because some participants found the cap of the gel bottle difficult to open due to finger stiffness. No evidence of damage to skin integrity was observed. Overall, skin moisture content had improved by the end of the study. Residents preferred either of the test products to the liquid formulation currently in use by the LTCF.

**Conclusions:**

Overall, the elderly were willing to use ABHR for hand hygiene. Both products were well tolerated and preferred over the usual product provided by the LTCF. However, forgetfulness and difficulty rubbing the product over the hands due to finger stiffness posed a challenge for some residents. This could be overcome by using healthcare worker-assisted hand hygiene at specified times each day and prompts to serve as reminders to perform hand hygiene.

## Background

Elderly residents in long-term care facilities (LTCFs) are at increased risk of colonization or infection with multi-drug resistant organisms (MDROs) [1, 2]. Infections caused by MDROs result in increased morbidity and mortality, frequent hospitalization and an increased demand for treatment using newer, more expensive antibiotics [2]. Residents of LTCFs are recognized as an important reservoir of MDRO in hospital settings [3, 4]. Hospitalization, in turn, may lead to colonization of previously uncolonized residents who then carry the MDRO to the home upon discharge [3]. Colonized or infected residents may have MDRO contamination of their hands [5]. Such hand contamination could lead to cross-transmission by direct contact with staff of the home or other residents [5], or indirectly via environmental contamination [2, 6]. In a study involving elderly patients at a post-acute care facility, 11% had methicillin-resistant *Staphylococcus aureus* (MRSA), and 13.7% vancomycin resistant enterococcal contamination of their hands [7]. In a separate study of MRSA nasal carriage, 95% (40/42) of elderly LTCF residents with proven nasal carriage, were also culture positive for hand contamination with MRSA [8]. Organisms such as MRSA and vancomycin resistant enterococci (VRE) can be transmitted indirectly via the environment [9]. A study conducted in a veterans affairs nursing home in the US noted that direct contact between residents and staff occurred frequently in the common areas of the facility outside of the resident’s room (5.8 times per hour), while contact between residents and the environment occurred 12.2 times per hour and this occurred most often in the dining room [10]. This study did not include contact between staff and patients during patient care procedures.

In the past, the focus was mainly on attempts to improve healthcare worker hand hygiene compliance with sometimes limited success [11]. More recently, the role of the patient’s hands in transmission of infection is receiving increased attention [12, 13] and efforts have been made to improve patient hand hygiene, particularly in the acute care setting [14].

Improving hand hygiene in elderly residents in LTCF could be an important infection control measure that would relieve the burden of healthcare associated infections. Although hand-washing facilities are provided in all LTCFs, these may not be readily accessible for many elderly residents if they have limited mobility. Alcohol-based hand rub (ABHR) is usually provided but few studies have investigated acceptability of ABHR for hand hygiene amongst this group. We aimed to investigate the acceptability and tolerability of two ABHRs for elderly residents in long-term care using a modified version of the World Health Organisation (WHO) Protocol Method 2 [15] for evaluation of two or more hand hygiene products.

## Methods

### Setting

This was a crossover study involving a convenience sample of elderly participants from a LTCF for the elderly in Hong Kong. The study was performed between February and March 2016.

Existing practices in the LTCF did not focus on elderly hand hygiene. Dispensers of ABHR (500 mL volumes of liquid rinse) were wall-mounted at a few sites in the home but most hand rub was placed at the nurses’ station and the emphasis was placed on staff hand hygiene.

### Study design

The study was based on a modified version of the WHO published protocol ‘Method for evaluation and comparison of tolerability and acceptability of different alcohol-based hand rubs: Method 2’ [15]. This method is used for the evaluation of two or more ABHRs. Since this protocol was originally designed for healthcare workers, we removed some questions that would not apply to our target group of elderly residents. Subjects were not asked to stop using hand care products during the study period. Questions on whether the subject suffered from irritative or atopic dermatitis, rhinitis/allergic conjunctivitis, asthma, or intolerance to alcohol were omitted from the checklist and instead the information for each participant was obtained from the staff of the home. Any subject suffering from any of these conditions was excluded from the study. We inserted a question on product preference at the end of the checklist. The modified checklist as well as rationale for modification/deletion of some questions are available as Additional file 1: Table S1 (a) and Additional file 2: Table S1 (b) respectively.

The modified checklist was translated to Chinese and back translated to English by a separate translator to confirm accuracy of the translation. The finalized checklist was administered to ten elderly residents of a separate LTCF to check their understanding of the questions and ability to answer.

The nurse in charge at the LTCF assisted in identifying 36 residents that were willing to participate in the study, and were capable of providing informed consent, and answering the questions from the WHO checklist.

**Fig. 1:**
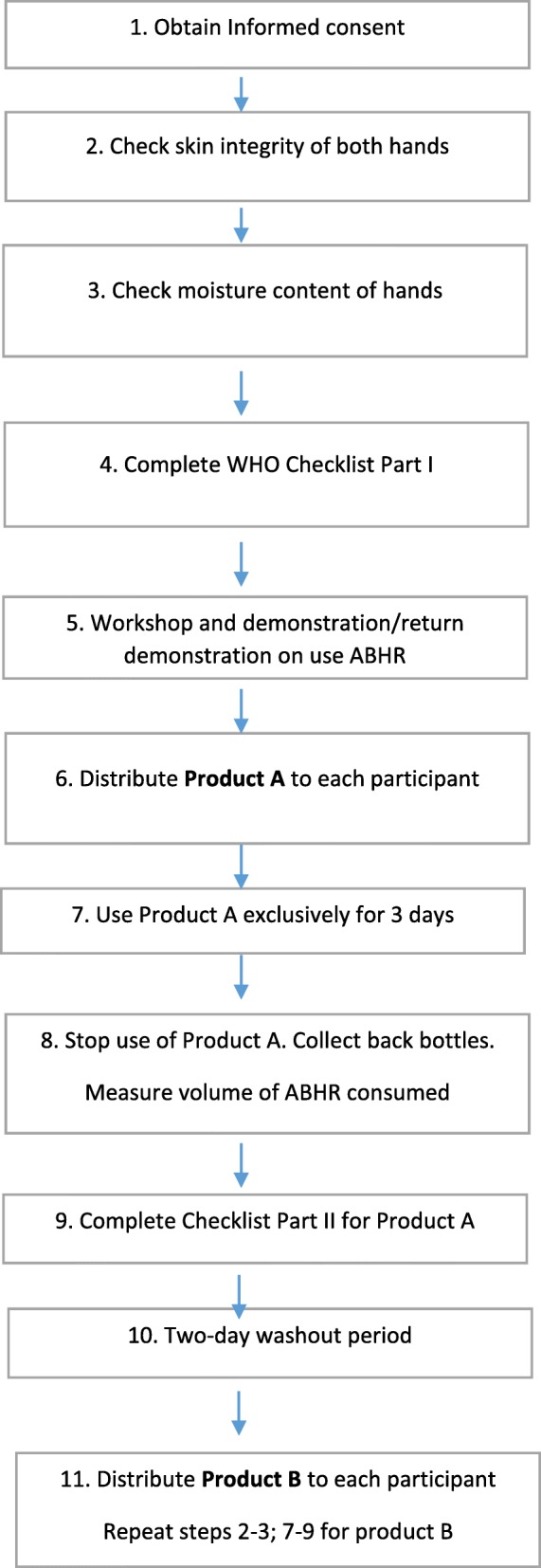
Flow chart of the protocol conducted to determine acceptability and tolerability of two ABHR hand hygiene products

Prior to distributing the first ABHR, all participants attended a workshop demonstrating correct use and benefits of ABHR for hand hygiene. A nurse then assessed the skin condition of all subjects [15]. Participants were asked to use each of two ABHRs (Product A and B) exclusively for hand hygiene over a three-day period. A portable bottle of Product A was then distributed to all participants. A two-day washout period was imposed before switching to Product B. Subjects were requested to avoid the use of other ABHR for the duration of the study. A flow chart outlining the protocol used is provided in Fig. 1.

### Products A and B

Product A was a gel (Microshield Angel blue antimicrobial hand gel; www.schuelke.com) which contains 70% ethanol plus a moisturizer and emollient. It has a light fragrance and is blue in colour. A pocket-sized bottle (125 ml volume) with a reclosable cap was distributed to participants. Product B was a fragrance-free foam with emollient (Avagard™; 3 M™). The active ingredient is 70% v/v ethanol. The portable version is provided in 50 mL volumes in a pushdown, lightweight pump bottle.

The accompanying instructions for both products required application of sufficient product to thoroughly wet all surfaces of the hands and fingers and then rub for at least 30 s or until dry. To determine the volume of ABHR used during the test periods, the bottles were weighed before distributing and again at the end of the test period.

### WHO checklist

The checklist [15] consisted of two parts (Questionnaire Parts I and II). Part I gathered information on demographics as well as current practices with respect to hand hygiene and skin care. This was completed once only for each participant prior to commencing the testing phase. Part II investigated acceptability and tolerability of each ABHR and was completed at the end of Day 3 for each product tested. All checklists were completed in a communal room provided by the LTCF. Where necessary, assistance in answering the questions was provided for subjects by research study staff.

### Determination of skin integrity and moisture content

The research nurse evaluated the skin health of the hands of participants for redness, scaliness, fissures or any other evidence of a skin reaction to the test products. The moisture content was determined using a Scalar moisture checker probe (Scalar moisture checker for skin, Science Technology Resources, Ca, USA). This was applied to the skin using gentle pressure and a digital reading recorded. Moisture content checking was performed in duplicate and a mean value determined. The probe was decontaminated using an alcohol wipe before proceeding to the next participant. The same site on the back of the hand was tested for each subject to ensure consistency of sampling.

### Workshop on use of ABHR

A workshop and training session were provided by the research nurse to demonstrate correct use of ABHR by elderly residents and explain the benefits of using ABHR for hand hygiene. Participants were particularly encouraged to use ABHR before each of the main mealtimes but to wash their hands if they were visibly soiled. Since many residents may find it difficult to remember all seven steps for hand hygiene [16], we used the simplified three-step protocol of Tschudin-Sutter et al. [17]. Participants were instructed to apply a sufficient volume of ABHR to fill the palm of one cupped hand and then Step 1: rub hands together carefully; Step 2 rub the fingertips of one hand in the palm of the other to the best of their ability, and finally Step 3 perform rotational rubbing of both thumbs. Emphasis was placed on Step 1. Participants continued to rub hands together until the hands felt dry [17].

All subjects were given the opportunity to practice using the ABHR during the workshop and repeated demonstration and return demonstration was provided. Healthy elderly volunteers from our university’s Institute for Active Aging also attended the workshop as ambassadors for hand hygiene. They demonstrated hand rubbing with ABHR and encouraged participants to practice. Each subject was given a personal portable bottle of ABHR (Product A) at the end of the workshop. Leaflets with a simple diagram of instructions on use of ABHR were distributed to each elderly subject to serve as a reminder of technique.

During the test period, a schedule was created to ensure that elderly ambassadors, student nurse helpers, and/or the research nurse, visited the LTCF daily to check on participants’ compliance with use of ABHR, remind them to use their ABHR, and ensure there was no skin irritation or other problems arising during the test period. Any elderly subject observed to have difficulty manipulating the ABHR bottles or performing the hand rubbing action, was assisted by nursing student helpers who used gloved hands to help rub the product into the hands.

### Data analysis

All data were entered for analysis into SPSS version 23. To comply with the WHO protocol, descriptive statistics were used for results presentation. Evaluation of tolerability and acceptability of both ABHRs was performed according to the WHO protocol [9]. In Questionnaire Part II: “Evaluation of the Test Product” the items colour and smell (fragrance) must score > 50% above four in the seven-point scale while all other items (texture, irritation, drying effect, etc.) must score > 75% above four. When presenting the results of the seven-point Likert scale used to determine product acceptability, scores were pooled as follows: 1–3 (low rating); 4–5 (neutral); 6–7 (highly rated) for data analysis purposes. Data on skin moisture content was interpreted following manufacturer’s instructions (Additional file 2: Table S2) for interpretation of readings for the Scalar moisture checker probe.

## Results

Overall, 36 residents participated in the study. Demographic details and factors influencing skin tolerance to ABHR are shown in Table 1. Although residents came together at mealtimes, their assigned rooms were spread over five floors of the LTCF; 12 residents (33%) were located on the first floor; 14 (39%) on the second; eight (22%) on the fourth floor and one resident each (2.8%) on the third and fifth floors. The mean age was 85 years (standard deviation ±8; range 70–98); 25 (70%) were female. Almost 31% of participants claimed to use hand lotion at least once per day (36% of females; 18% of males). The majority of participants (61%) thought they could improve their own hand hygiene. A large proportion (68%) thought that forgetfulness, but not a lack of time, nor the possibility of damage to skin, could hinder their ability to use ABHR for hand hygiene (Table 1).

**Table 1:** Subject demographics and responses to the WHO checklist for hand hygiene product tolerability and acceptability

Demographics	Age range	Frequency (%)			
	70–75	4 (11.1)			
	76–80	4 (11.1)			
	81–85	6 (16.67)			
	86–90	12 (33.33)			
	91–95	8 (22.27)			
	> 95	1 (2.78)			
	Missing	1 (2.78)			
Mean ± SD in years	85 ± 8.25				
Sex	Female	25 (69.4)			
	Male	11 (30.6)			
Evaluation of factors influencing skin tolerance			Response Options		
Use of a protective hand lotion/cream (outside of the test period)			As often as possible		1 (2.8)
			Several times per day		6 (16.7)
			Once per day		4 (11.1)
			Sometimes		1 (2.8)
			Rarely		1 (2.8)
			Never		23 (63.9)
Do you think you can improve your own hand hygiene compliance?			Yes		22 (61.11)
			No		5 (13.89)
			Perhaps		7 (19.44)
			Missing		2 (5.56)
It may be difficult for you to use an alcohol-based hand hygiene product because of:		Always	Sometimes	Never	Missing
	Forgetfulness	6 (16.67)	17 (47.22)	11 (30.56)	2 (5.56)
	Lack of time	1 (2.78)	0	33 (97.1)	2 (5.56)
	Damaged skin	1 (2.78)		33 (91.67)	2 (5.56)

**Fig. 2:**
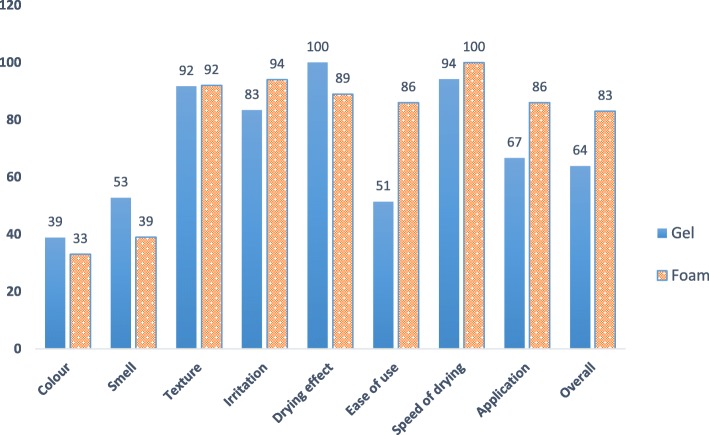
User acceptability for each of the ABHR products tested*

Both tested ABHRs passed the WHO criteria for product tolerability and acceptability (Table 2). The majority of residents thought that both ABHRs dried quickly and that neither product felt sticky. More than 90% reported no drying effect on the hands. There was a notable difference in ratings for ‘ease of use’ (Fig. 2) with the majority of subjects (86%) finding the foam pump easier to manipulate than the portable bottle of gel (51%). A similar result was observed for “application”; 86% of participants thought the foam was pleasant to apply while only 67% rated application of the gel as pleasant. All participants reported no irritation when using the foam but 14% thought the gel caused irritation. Overall, participants were very satisfied with both ABHRs (Table 2) but rated the foam more highly (83%) than the gel (64%).

**Table 2:** Evaluation of acceptability of both ABHR test products according to WHO criteria^#^

Variable	Rating	Product A (Gel)		Product B (Foam)^	
		N	%	N	%
Colour	Pleasant	14	38.89	12	38.33
	Neutral	22	61	22	61.11
	Missing	0	0	2	5.56
Smell	Pleasant	19	52.78	14	38.89
	Neutral	16	44.4	20	55.56
	Unpleasant	1	2.78	0	0
Texture	Not sticky at all	33	91.67	33	91.67
	Neutral	2	5.55	1	2.78
	Sticky	1	2.77	0	0
	Missing	0	0	2	5.56
Irritation	Not irritating	30	83.33	34	94.44
	Neutral	1	2.77	0	0
	Irritating	5	13.88	0	0
	Missing	0	0	2	5.56
Drying effect	Not at all	33	91.7	32	88.89
	Some	2	5.6	1	2.78
	A lot	1	2.8	1	2.78
	Missing	0	0	2	5.56
Ease of use	Very easy	18	51.43	31	86.11
	Neutral	9	25	3	8.33
	Very difficult	8	22.2	0	0
	Missing	1	2.78	2	5.56
Speed of drying	Fast	32	88.89	34	94.44
	Medium	2	5.56	0	0
	Missing	2	5.56	2	5.56
Application	Pleasant	24	66.67	31	86.11
	Neutral	12	33.3	3	8.33
	Missing	0	0	2	5.56
Overall	Very satisfied	23	63.89	30	83.33
	Neutral	13	36.1	4	11.11
	Missing	0	0	2	5.56

^**#**^WHO criteria for product acceptability: Items Colour & Fragrance (smell): must achieve a rating of ≥50% above 4 (neutral to very satisfied). All other items: ≥75%. Both ABHR products tested fulfilled the criteria

^Two subjects were hospitalized during the washout period and were unable to participate in testing of product B

**Fig. 3:**
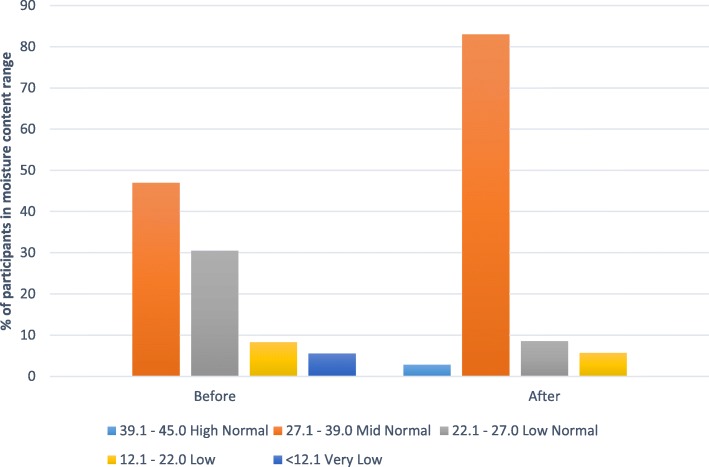
Comparison of participants’ skin moisture content before and after the testing period

Data on frequency of hand hygiene practices is shown in Table 3. After completion of the first test period (Product A, gel), 50% of participants thought that the study had changed their hand hygiene practices. By the end of the study, this figure increased to 69%. Half of the participants (50%) claimed to practice hand hygiene at least 1–5 times per day during the test period. Participants (42%) noted a major difference between Product B (foam) and the product normally used in the LTCF (Table 4) and at least 50% preferred the test products to their usual product (69% for the foam). One-third of subjects (33%) thought that the gel could improve their hand hygiene compliance while 75% thought that use of the foam could improve compliance. Overall, 53% preferred the foam to the gel for hand rubbing. The volume of ABHR used was low: assuming a minimum volume of 1.5 mL of ABHR should be used during each hand hygiene opportunity, 4.5 mL would be consumed daily if hand rubbing was performed three times per day. However, only 36% (13/36) of residents used > 6 ml of product (data not shown). Despite this, there appeared to be an overall improvement in skin moisture content (Fig. 3); 47% of subjects were in the mid-normal range at commencement of the study and this increased to 83% by the end of the study (Fig. 3). There was no evidence of damage to skin integrity caused by either product and in the subjective evaluation of skin assessment; more than 90% of residents rated their skin integrity as perfect following use of either test product (Table 5).

**Table 3:** Evaluation of frequency of hand hygiene practices

		Product A (Gel)	Product B^ (Foam)
(a) In what percentage of times where hand hygiene is recommended, did you really clean your hands?	Variable	N (%)	N (%)
	0–10%	11 (30.56)	3 (8.33)
	20–40%	12 (33.33)	12 (33.33)
	50–70%	6 (16.67)	14 (38.89)
	80–100%	7 (20.58)	5 (13.89)
	Missing	0	2 (5.56)
(b) Has the present study changed your hand hygiene practice?	Yes	18 (50%)	25 (69.44)
	No	18 (50%)	9 (25)
(c) During your last five opportunities for hand hygiene, how many times did you use handrubbing to clean your hands?	0	1 (2.78)	0
	1	11 (30.56)	10 (27.75)
	2	4 (11.11)	13 (36.11)
	3	7 (19.44)	4 (11.11)
	4	3 (8.33)	4 (11.11)
	5	10 (27.78)	3 (8.33)
	Missing	0	2 (5.56)
	< 1	1 (2.78)	0
(d) On average, how often did you practice hand hygiene each day (during the test period)	1–5	18 (50)	18 (50)
	6–10	17 (47.22)	15 (41.67)
	11–15	0	1 (2.75)
	> 15	0	0
	Missing	0	2 (5.56)

^Two subjects were hospitalized during the washout period and were unable to participate in testing of product B

**Table 4:** Evaluation of test products and product preference

Question	Response options	Product A		Product B^	
		N	%	N	%
Are there differences between the test product and the product used in this elderly home?	Major difference	4	11.11	15	41.67
	Some	18	50	18	50
	None	14	38.89	1	2.78
	Missing	0	0	2	5.56
Which product do you prefer?	Test product	18	50	25	69.44
	Usual product of (RCHE)	3	8.33	2	5.56
	No difference	15	41.67	7	19.44
	Missing	0	0	2	5.56
Do you think that the test product could improve your hand hygiene compliance?	Yes absolutely	12	33.33	27	75
	No	5	13.89	1	2.78
	Neutral	19	52.78	6	16.67
	Missing	0	0	2	5.56
Did you have any preference between the test products used?^#^					
Gel	0	0			
Foam	19	52.78			
No preference	15	41.67			
Missing	2	5.56			

# This question was asked at the end of the testing period (*N* = 34)

^Two subjects were hospitalized during the washout period and were unable to participate in testing of product B

**Table 5:** Evaluation of skin condition

D. Evaluation of skin condition		Product A		Product B^	
Self-assessment of the skin of your hands (after use of the test product)		N	%	N	%
	Rating				
Appearance	Normal	35	97.22	33	91.66
	Neutral	1	2.78	1	2.78
	Missing	0		2	5.56
Intactness	Normal	34	94.4	34	94.4
	Neutral	2	5.56	0	0
	Missing	0		2	5.56
Moisture content	Normal	34	94.4	34	94.4
	Neutral	2	5.56	0	0
	Missing	0	0	2	5.56
Sensation	Normal	34	94.4	34	94.4
	Neutral	2	5.56	0	0
	Missing	0	0	2	5.56
Overall integrity of the skin of your hands	Perfect	33	91.66	34	94.4
	Neutral	3	8.33	0	0
	Missing	0	0	2	5.56

^Two subjects were hospitalized during the washout period and were unable to participate in testing of product B

During the study, we observed that some participants were forgetful and seven (19%) misplaced their personal bottles of ABHR (Product A). A few subjects suffered from joint stiffness and were unable to rub the product over the entire hands as instructed.

## Discussion

We aimed to determine acceptability and tolerability of two ABHRs for use by elderly residents in long-term care using the WHO protocol Method 2 [16]. We selected one gel and one foam as these formats should be easier to apply, have less drying effect, and be less likely to accidentally spill during use [18]. In the past, doubts had been cast over the efficacy of ABHR gels and foams for hand hygiene [19–21], but more recent studies have concluded that there is no difference in efficacy between new generations of gel or foam formats and liquid (rinse) ABHRs when appropriately applied [22, 23]. To the best of our knowledge, this is the first time the WHO protocol has been used for this demographic.

Our results revealed that the elderly residents preferred both of the test products to the usual one used by the home, which was a liquid rinse formulation containing glycerol as humectant. When comparing test products, there appeared to be a preference for the foam. This is consistent with the findings from some studies investigating preference of ABHR formulation by patients in acute care settings as well as for healthcare workers [11, 24]. Additionally, the foam in our study was provided in a pushdown pump which residents found easier to handle. Product acceptability, including ease of use, is essential in order to ensure user compliance [25]. Subjects in our study found the portable bottles of gel with plastic caps difficult to manipulate. This may explain why this product was rated lower overall than the foam with some elderly misplacing their bottles of gel during the test period.

Product tolerability and skin compatibility are also critical, and studies have demonstrated that a product that is not well tolerated will not be well-accepted [25]. In our study, objective assessment by an independent observer as well as subjective assessment by users demonstrated that both products were well tolerated.

Some of the elderly residents in our study could not perform the hand hygiene steps precisely due to stiffness in their joints or having deformed fingers due to arthritis. Providing individual bottles of ABHR to this group may not lead to improvements in hand hygiene. Even though subjects were deemed cognitively sound and capable of participating in the study, forgetfulness was an issue for a few subjects. Therefore, assistance with hand rubbing would be required for specific residents in order for any intervention to be successful. This could involve the staff of the home placing 500 mL pump dispensers in communal dining tables immediately before each of the three main mealtimes and prompting residents to perform hand hygiene before dining. ABHR could be dispensed on the hands and gently rubbed over the hands for those requiring assistance, while those capable of performing hand rubbing by themselves could proceed to do so. This strategy could overcome many of the difficulties experienced by providing individual portable bottles to residents and should not put undue burden on the healthcare workers and healthcare assistants of the home. An additional advantage would be the ability to include subjects that are not cognitively sound and who would not normally be able to use ABHR. Interventions for improving hand hygiene in the elderly have been attempted with some success. Schweon et al. (2013) implemented a programme to improve hand hygiene for both staff and residents of a skilled nursing facility in the US and were able to demonstrate reductions in rates of lower respiratory tract infections and surgical site infections over the 22-month period of the study [11]. However, they did not monitor compliance to hand hygiene by residents and did not provide suggestions for long-term sustainability of the intervention [11]. In a separate study, efforts to improve hand hygiene of residents in a LTCF involved a multidisciplinary team to design and implement an intervention that would encourage hand hygiene by residents at mealtimes [26].

In this study, we used a simplified hand rubbing technique based on the three-step method described by Tschudin-Sutter et al. [17] since it is easier to perform and could potentially improve compliance. This method was demonstrated to be as effective as the WHO six-step method for reduction of bacterial counts on the hands of healthy volunteers [17]. Importantly, although named “a three-step method”, this simplified technique nevertheless requires the covering of all hand surfaces with ABHR, and subsequent rubbing of all hand surfaces, essentially applying the originally described WHO six-step technique, but condensing this to three steps [17].

This study had some limitations. The WHO protocol used [15] was designed for healthcare workers, but it was not possible to use randomization and blinding in our study due to the limitations of the setting. Additionally, since both ABHRs used were commercial preparations, it was not possible to conceal the product type, one being a gel in a portable bottle, the second being a foam pump. Elderly LTCF residents would not be expected to perform hand hygiene as frequently as healthcare workers; thus, we did not insist on use of 30 mL of product per day. Subjects required assistance in completing the questionnaires. It is possible that they gave the responses they thought would be most appropriate or acceptable to the research nurse. However, there was clearly a difference in responses when determining acceptability of the gel in comparison to the foam ABHR. We did not strictly follow the WHO scale for evaluation of skin condition by an objective assessor, however, the research nurse was able to objectively evaluate skin condition and subjective evaluation ratings were very high. Additionally, many studies have demonstrated that gel and foam ABHRs are gentle on the hands. Finally, participants’ acceptability of both products was excellent. It is likely that acceptability could not be achieved if skin tolerability was a problem.

## Conclusion

Overall, the elderly residents in our study were willing and even enthusiastic about using ABHR for hand hygiene. However, supervision and assistance from the staff of the home would be required to prompt them to clean their hands and to assist those whose hands are too stiff to rub the product over the entire hands. Encouraging hand hygiene three times per day during mealtimes should be feasible and sustainable and would not require additional staffing. The majority of participants favoured the foam product and this may be important when attempting to encourage compliance to hand hygiene. Both ABHRs tested fulfilled the WHO criteria for tolerability and acceptability. Given the critical issue of multi-drug resistance, the reservoirs of MDROs in residential care settings for the elderly, and the increased staff to resident care often required for elderly with co-morbid conditions, it is essential that efforts to improve hand hygiene in this group be attempted. Improved hand hygiene could reduce the transmission of healthcare associated pathogens and relieve demand on our already dwindling supply of antibiotics.

## Supplementary information

**Additional file 1:** **Tabel S1.** (a) Protocol for Evaluation and Comparison of Tolerability and Acceptability of Different Alcohol-based Handrubs: Method 2 (modified) –word document.

**Additional file 2:** **Table S1.** (b) Modifications to the WHO checklist for evaluation and comparison of tolerability and acceptability of different alcohol based hand rubs: Method 2, with justification for changes made. –word document. **Table S2.** Skin Moisture content-interpretation of readings for the Scalar moisture checker probe.

## Data Availability

The datasets used and/or analysed during the current study are available from the corresponding author on reasonable request.

## Abbreviations

ABHR: Alcohol-based hand rub
LTCFs: Long-term care facilities
MDROs: Multi-drug resistant organisms
MRSA: Methicillin resistant *Staphylococcus aureus*
VRE: Vancomycin resistant enterococci
WHO: World Health Organization
