# Supplementary material for: Acceptability and tolerability of alcohol-based hand hygiene products for elderly residents in long-term care: a crossover study
Source: Antimicrob Resist Infect Control. 2019 Oct 29;8:165. doi: 10.1186/s13756-019-0610-7 (PMC6819464; doi:10.1186/s13756-019-0610-7)
Supplement: Supplementary file 1 — Additional file 1: Tabel S1. (a) Protocol for Evaluation and Comparison of Tolerability and Acceptability of Different Alcohol-based Handrubs: Method 2 (modified) –word document. [file 13756_2019_610_MOESM1_ESM.docx]

**Tabel 1(a) Protocol for Evaluation and Comparison of Tolerability and Acceptability of Different Alcohol-based Handrubs: Method 2 (modified)**

Questionnaire – Part 1

(To be completed **once** **only** per participant)

| Participant no: |  |
| --- | --- |
|  |  |
| Date of questionnaire's return: (day / month / year) |  |

Evaluation of factors influencing skin tolerance

**Age:**

**Sex**:  F  M

**Do you normally use a protective hand lotion/cream (outside of the test period)?**

As often as possible  Several times/day  Once/day

Sometimes, depending on the season  Rarely  Never

**Evaluation of frequency of hand hygiene practices**

**Do you think you can improve your own hand hygiene compliance?**

Yes  No  Perhaps

**It may be difficult for you to use an alcohol-based hand hygiene product because of:**

***Forgetfulness***  Always Sometimes Never

***Lack of time*** Always Sometimes Never

***Damaged skin*** Always Sometimes Never

Questionnaire – Part 2

| Product type (A or B) |  |  | Amount of Product used (ml) |  |
| --- | --- | --- | --- | --- |

Evaluation of frequency of hand hygiene practices

**In what percentage of times where hand hygiene is recommended, do you really clean your hands?**

0%  10%  20%  30%  40%  50%  60%  70%  80%  90%  100%

**Has the present study changed your hand hygiene practice?**

Yes  No

**During your last five opportunities for hand hygiene, how many times did you use handrubbing to clean your hands?**

0  1  2  3  4  5

**On average, how often did you practise hand hygiene each day (during the test period)?**

< 1  Between 1 and 5  Between 6 and 10  Between 11 and 15  > 15

Evaluation of the test product

**What is your opinion of the test product for hand hygiene?**

***Colour***  Unpleasant ^- - -^ ^- - -^ ^- - -^^- - -^ ^- - -^ ^- - -^  Pleasant

***Smell*** Unpleasant ^- - -^ ^- - -^ ^- - -^ ^- - -^^- - -^ ^- - -^  Pleasant

***Texture*** Very sticky ^- - -^ ^- - -^ ^- - -^ ^- - -^^- - -^ ^- - -^  Not sticky at all

***Irritation (stinging)*** Very irritating ^- - -^ ^- - -^ ^- - -^ ^- - -^^- - -^ ^- - -^  Not irritating

***Drying effect*** Very much ^- - -^ ^- - -^ ^- - -^ ^- - -^^- - -^ ^- - -^  Not at all

***Ease of use*** Very difficult ^- - -^ ^- - -^ ^- - -^ ^- - -^^- - -^ ^- - -^  Very easy

***Speed of drying*** Very slow ^- - -^ ^- - -^ ^- - -^ ^- - -^^- - -^ ^- - -^  Very fast

***Application*** Very unpleasant ^- - -^ ^- - -^ ^- - -^ ^- - -^^- - -^ ^- - -^  Very pleasant

***Overall evaluation*** Dissatisfied ^- - -^ ^- - -^ ^- - -^ ^- - -^^- - -^ ^- - -^  Very satisfied

**Are there differences between the test product and the product used in this elderly home?**

Major ^- - -^ ^- - -^ ^- - -^ ^- - -^^- - -^ ^- - -^  No

**Which product do you prefer?**

Usual product  Test product  No preference

**Do you think that the test product could improve your hand hygiene compliance?**

Yes, absolutely ^- - -^ ^- - -^ ^- - -^^- - -^ ^- - -^ ^- - -^  Not at all

Evaluation of skin condition

**Self-assessment of the skin on your hands (after use of the test product):**

**Appearance**

***(supple, red, blotchy, rash)*** Abnormal ^- - -^ ^- - -^ ^- - -^^- - -^ ^- - -^ ^- - -^  Normal

***Intactness (abrasions, fissures)*** Abnormal ^- - -^ ^- - -^ ^- - -^^- - -^ ^- - -^ ^- - -^  Normal

***Moisture content (dryness)*** Abnormal ^- - -^ ^- - -^ ^- - -^^- - -^ ^- - -^ ^- - -^  Normal

***Sensation (itching, burning, soreness)*** Abnormal ^- - -^ ^- - -^ ^- - -^^- - -^ ^- - -^ ^- - -^  Normal

**How would you assess the overall integrity of the skin on your hands now?**

Very altered ^- - -^ ^- - -^ ^- - -^ ^- - -^^- - -^ ^- - -^  Perfect

**To be asked only after Product B testing completed**

**Did you have a preference for either of the test products? Product A (gel)** ^- -^ **Product B (foam)**  ^-^ ^- - -^ **No preference**

Thank you for your participation!
