# Supplementary material for: Acceptability and tolerability of alcohol-based hand hygiene products for elderly residents in long-term care: a crossover study
Source: Antimicrob Resist Infect Control. 2019 Oct 29;8:165. doi: 10.1186/s13756-019-0610-7 (PMC6819464; doi:10.1186/s13756-019-0610-7)
Supplement: Supplementary file 2 — Additional file 2: Table S1. (b) Modifications to the WHO checklist for evaluation and comparison of tolerability and acceptability of different alcohol based hand rubs: Method 2, with justification for changes made. –word document. Table S2. Skin Moisture content-interpretation of readings for the Scalar moisture checker probe. [file 13756_2019_610_MOESM2_ESM.docx]

**Supplementary Table 1(b).**

Modifications to the WHO checklist for evaluation and comparison of tolerability and acceptability of different alcohol based hand rubs: Method 2, with justification for changes made.

| **Questionnaire Part I** | |
| --- | --- |
| **Original question** | **Modifications and Justification** |
| Professional group | All participants were elderly ethnic Chinese residing in a residential care home for the elderly. Items removed. |
| Questions on skin colour |  |
| Climate and season | The setting was Hong Kong, which is sub-tropical. The study was performed in the months of Feb –Mar 2016. The average temperature at that time was 19.55°C and the humidity was 66.44%. |
| Questions on work related activities:   - Professional group; - Use of alcohol hand rub at work; - Working full time or part time. | Not relevant for this setting |
| Whether the subject suffers from:   - Irritative or atopic dermatitis; - Rhinitis/allergic conjunctivitis; - Asthma; - An intolerance to alcohol. | These were deleted from the checklist and instead the information for each participant was obtained from the staff of the home  Any subject suffering from any of these conditions was excluded from the study. |
| It may be difficult for you to use an alcohol-based hand hygiene product because of: | The seven checkboxes for frequency were removed and replaced with Always Sometimes Never.  Prior to commencing the study, the checklist was tested on a group of 10 elderly to check their understanding of the questions and ability to answer. Subjects found the seven checkboxes confusing ad were more comfortable when provided with the three options: Always Sometimes Never |
| **Questionnaire Part II** | |
| Direct contact with patients | Not a relevant question for this subject group |
| On average, how often do you practice hand hygiene during a working hour (during the test period) | This was changed to:  On average, how often did you practice hand hygiene during the day (during the test period) |
| Are there differences between the test product and the product used in your hospital? | Are there differences between the test product and the product used in this elderly home? |
|  |  |
| This question was inserted at the end of testing Product B | To be asked only after Product B testing completed  Did you have a preference for either of the test products?  Product A (gel) Product B (foam) No preference |

Supplementary Table 2. Skin Moisture content-interpretation of readings for the Scalar moisture checker probe.

| **Reading** | **Indication** | **Recommendation** |
| --- | --- | --- |
| 45.1 and up | Very High | Use moisturizer as needed |
| 39.1 - 45.0 | High Normal | Continue using moisturizer |
| 27.1 - 39.0 | Mid Normal | Use moisturizer to raise moisture levels |
| 22.1 - 27.0 | Low Normal | Moisturizer is recommended to raise moisture levels |
| 12.1 - 22.0 | Low | Moisturizer is highly recommended |
| <12.1 | Very Low | Moisturizer is needed |

|  |  |  |  |
| --- | --- | --- | --- |
|  |  |  |  |
|  |  |  |  |
